# Metabolic and Pharmacokinetic Profiling Studies of N, N-Dimethylaniline-Heliamine in Rats by UHPLC-Q-Orbitrap MS/MS

**DOI:** 10.3390/molecules29184324

**Published:** 2024-09-12

**Authors:** Ruqi Xi, Rahima Abdulla, Jurakulov Sherzod, Vinogradova Valentina Ivanovna, Maidina Habasi, Yongqiang Liu

**Affiliations:** 1 State Key Laboratory Basis of Xinjiang Indigenous Medicinal Plants Resource Utilization, CAS Key Laboratory of Chemistry of Plant Resources in Arid Regions, Xinjiang Technical Institute of Physics and Chemistry, Chinese Academy of Sciences, Urumqi 830011, China; xiruqi20@mails.ucas.ac.cn (R.X.); rahima@ms.xjb.ac.cn (R.A.); 2 S. Yu. Yunusov Institute of the Chemistry of Plant Substances, Academy of Sciences of the Republic of Uzbekistan, Tashkent 100170, Uzbekistan; j.sherzod.78@mail.ru (J.S.); 1946viv@mkail.ru (V.V.I.); 3 University of Chinese Academy of Sciences, No. 19 (A) Yuquan Road, Shijingshan District, Beijing 100049, China

**Keywords:** N, N-dimethylheliamine, pharmacokinetics, metabolites, UHPLC-MS/MS, rats, antiarrhythmic, XenoSite predicts, tetrahydroisoquinoline alkaloid

## Abstract

Cardiovascular disease is the first cause of death worldwide and kills more people each year than any other cause of death. N, N-dimethylaniline-heliamine (DH), a synthetic tetrahydroisoquinoline alkaloid, has shown notable antiarrhythmic activity. However, the metabolic processes and pharmacokinetic characteristics of DH in rats have not been studied. This study aims to identify its metabolites, as well as develop and validate a rapid and efficient bioanalytical method for quantifying DH in rat plasma over a wide range of concentrations. Its metabolites were characterized in silico, in vitro, and in vivo. A series of 16 metabolites were identified, of which 12 were phase I metabolites and 4 were phase II metabolites. A low probability of DH binding to DNA, protein, and glutathione is predicted by the in silico model. The main metabolic processes of DH were demethylation, dehydrogenation, glucuronidation, and sulfation. Concentration–time profiles were generated by analyzing the plasma, and the outcomes were analyzed via non-compartmental analysis to identify the pharmacokinetic parameters. Among the detected parameters were the volume of distribution, estimated at 126,728.09 ± 56,867.09 mL/kg, clearance at 30,148.65 ± 15,354.27 mL/h/kg, and absolute oral bioavailability at 16.11%. The plasma distribution volume of DH was substantially higher than the overall plasma volume of rats, which suggests that DH has a specific tissue distribution in rats. This study suggests that DH is appropriately bioavailable and excreted via a variety of routes and has low toxicity.

## 1. Introduction

**Figure 1:**
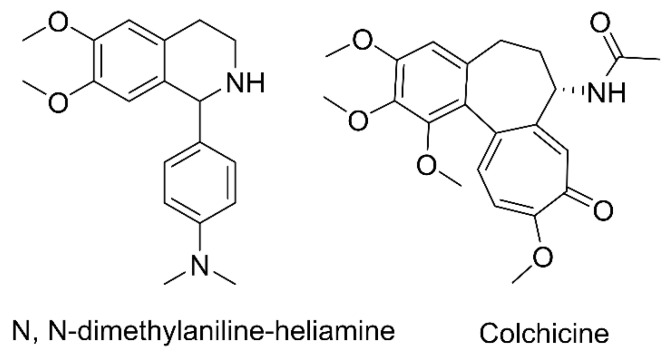
Chemical structures of DH and colchicine (IS).

Tetrahydroisoquinolines are spirocyclic molecules consisting of a benzene ring linked to an azepine ring [1]. The position of the hydrogen atoms on the benzene and aza heterocycles in the tetrahydroisoquinoline molecule is very specific and their arrangement determines the chemical properties and reactivity of the molecule [2]. It is found in many families of plants including magnoliaceae, camphoraceae, aristolochiaceae, ranunculaceae, berberidaceae, antihymenaceae, and populaceae [3]. Isoquinoline derivatives have a wide range of pharmacological actions, including antiarrhythmic, antibacterial, antiviral, hypotensive, and immunomodulatory, according to numerous investigations [4,5]. Some of the tetrahydroisoquinoline alkaloids such as berberine hydrochloride [6] and tetrahydropalmatine [7] are widely used in clinical practice. Therefore, a positive attitude towards the activity of alkaloids in living organisms is maintained. N, N-dimethylaniline-heliamine (DH) (Figure 1) is a synthetic tetrahydroisoquinoline derivative, and studies have shown that DH has promising antiarrhythmic activity [8]. Acute toxicity parameters of DH in rats are more than six times lower than those of verapamil [8]. Sarcoplasmic reticulum RyR_2_Ca^2+^ channels are inhibited by DH, which also acts as a membrane surface Na^+^–Ca^2+^ exchange inhibitor to decrease sarcoplasmic reticulum Ca^2+^ release [9].

Cardiovascular diseases account for the highest mortality rate worldwide, causing the loss of approximately 17.9 million lives annually. These ailments pertain to a set of heart and blood vessel disorders, comprising coronary heart disease, cerebrovascular disease, rheumatic heart disease, and various other related illnesses [10]. Arrhythmia is an abnormal excitation of the sinus node or one that arises outside the sinus node. This ultimately leads to slow, blocked, or abnormal conduction of excitation through abnormal channels [11]. Nearly 90% of acute cardiac deaths are caused by arrhythmia, which seriously threatens the life and health of the global population [4,12]. Arrhythmia treatment is divided into surgical treatment and medical treatment options. The surgical therapy options include electrical defibrillation, electrical cardioversion, implantable cardioverter-defibrillators, cardiac pacing, and catheter-based radiofrequency ablation [5,13]. Despite the diversity of surgical treatment options, patients have worries about capsular bag infection, heart perforation, and pacemaker electrode dislodgement [14,15]. Medical therapy options include (1) sodium channel blockers: quinidine [16], propafenone hydrochloride [17], etc.; (2) β blockers: metoprolol [18], propranolol [19], etc.; (3) potassium channel blockers: amiodarone, dofetilide, etc.; and (4) calcium channel blockers: verapamil [20], diltiazem [21], etc. The clinical classification of medicines is diverse and numerous (William’s classification [22], Sicilian classification [23], and modern classification [24]). However, the tendency to cause arrhythmias and the narrow therapeutic window limit the use of these medicines in clinical practice [25]. Therefore, it is necessary to discover medicines with a wider therapeutic window and progress their preclinical studies. Although DH (Figure 1) exhibits strong antiarrhythmic activity, its metabolic processes and pharmacokinetic characteristics in rats have not been studied.

The conventional isolation and purification of identified compounds require a sufficiently large amount of compound and a high level of purity [26]. However, the medicine is metabolized by CYP 450 enzymes in rats to produce a variety of metabolites at very low concentrations [27]. Consequently, the metabolites cannot be obtained as monomers by conventional isolation and purification. The high sensitivity and specificity of mass spectrometry combined with the high separation efficiency of the upper liquid phase greatly facilitates the study of medicine metabolism [28]. The objective of this research was to utilize in silico methods to anticipate the metabolic reactivity and sites of DH with DNA, proteins, and glutathione, followed by the in vitro (rat liver microsomes) and in vivo (plasma, urine, and feces) investigation of DH metabolites. This study was to create a dependable and sensitive bioanalytical technique for rat plasma analysis of DH. Ultimately, the metabolic pathways of DH were hypothesized, and the pharmacokinetic patterns of DH in rats were summarized.

## 2. Results and Discussion

### 2.1. UHPLC-MS/MS Condition

By experimenting with different ESI modes, DH was found to exhibit a stronger MS response in the positive ESI mode. The optimization process resulted in a method that achieved high sensitivity, short retention time, high resolution, and a symmetrical peak pattern. To further optimize the elution conditions, different organic solvents such as acetonitrile and methanol were evaluated. Considering that using methanol led to high column pressure and trailed sample peaks, the mobile phase was modified to acetonitrile. Additionally, a 0.1% formic acid aqueous phase modifier was implemented to promote ionization and ensure a stable pH value for the sample solution. Finally, an Acquity BEH C18 (2.1 × 75 mm, 1.7 µm particle size) column was chosen based on the above optimization results.

### 2.2. Metabolites of DH

**Figure 2:**
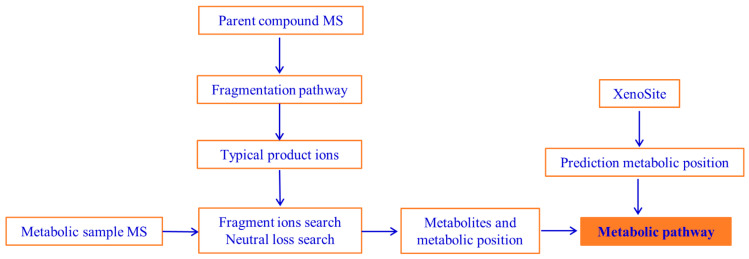
Schematic diagram of metabolites identification of DH.

It is vital for the pre-clinical determination of DH’s metabolic processes. Due to a handful of metabolites and the complex composition of feces, urine, and plasma, it is difficult to obtain the metabolites as monomers by purification. Firstly, the XenoSite was used to predict possible metabolic sites. Secondly, liver microsomes were employed to simulate metabolism and identify possible metabolites of DH. Finally, the metabolites of DH in feces, urine, and plasma were identified, and the translation process of DH was summarized. A schematic diagram of metabolite identification is shown in Figure 2. 

#### 2.2.1. Metabolic Site Prediction of DH

**Figure 3:**
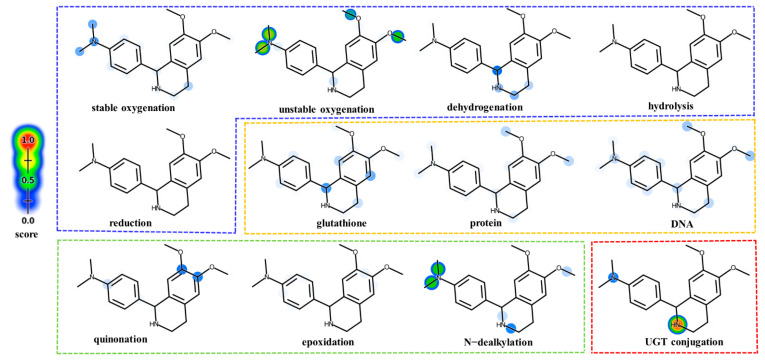
In silico prediction of phase I site (stable oxygenation, unstable oxygenation, dehydrogenation, hydrolysis, and reduction); site of reactivity (glutathione, protein, and DNA); site of quinonation, epoxidation, and N-dealkylation; site of glucuronidation in DH. Potential sites of metabolism are highlighted by a color gradient, distributed throughout a range of 0 (no color, minimal probability) to 1 (color, maximal probability).

The phase I metabolic model was trained with 20,736 humans in vitro and in vivo records. XenoSite displays the predicted results for 21 phase I metabolic reaction types on the following five molecular diagrams: stable oxygenation, unstable oxygenation, dehydrogenation, hydrolysis, and reduction. The prediction accuracy of the phase I model is 97.1% [29]. The model predictions showed that methyl was easily shed, and the tetrahydroisoquinoline ring was susceptible to oxidation. Medicinal chemists are committed to developing safe and effective medicines. However, unpredictable toxicity and adverse effects of the compounds themselves or their metabolites have resulted in 62% of compounds being withdrawn from the market or blacklisted [29,30]. Some electrophile groups in the structure of the compound can covalently bind to glutathione, proteins, and DNA leading to severe toxicity [31,32,33]. The prediction accuracy of the reactivity models was 90.8%, 94.9%, and 89.8% for glutathione, proteins, and DNA, respectively [24,25]. The model predictions indicate that DH has low glutathione, protein, and DNA binding capacity. Some molecules undergo epoxidation, quinonation, and N-demethylation metabolism to produce some electrophile groups. It can combine with glutathione, proteins, and DNA, and in some cases, it can cause an immune response leading to allergy. The accuracy of the epoxidation, quinonation, and N-demethylation models in XenoSite were 94.9%, 97.6%, and 97.0% [21,22,23], respectively. The model predictions display that DH demethylates readily form electrophile groups. Uridine diphosphate glucuronosyltransferases catalyze glucose binding to oxygen, nitrogen, sulfur, or carbon. It was easier for DH to be excreted, which reduced the toxicity of the DH accumulation in the body. The accuracy of the model’s predicted top1 and top2 sites bound to glucuronide was 86 and 97% [26], respectively. The results are included in Figure 3.

#### 2.2.2. Mass Fragmentation of DH

**Figure 4:**
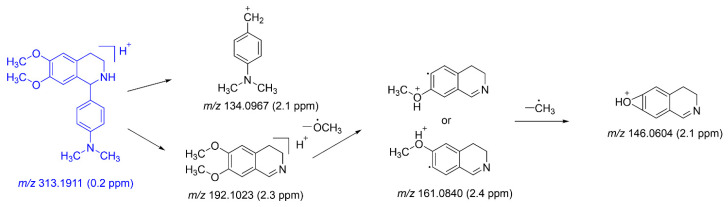
The characteristic fragments of the MS spectrum of DH.

As illustrated in Figure 4, the cleavage pattern of DH was analyzed. DH was positively identified using the product ion spectra of its reference standard, with the protonated molecular ion peak of DH registering at *m*/*z* 313.1911. A tetrahydroisoquinoline ring cleavage product was observed at fragment ion *m*/*z* 134.0967. Additionally, fragment ion *m*/*z* 192.1023 was generated from DH through the loss of a 4′-Dimethylaminophenyl, followed by the further loss of a methoxy radical to produce the product fragment ion at *m*/*z* 161.0840. Finally, fragment ion *m*/*z* 146.0604 was generated from the 161.0840 fragment ion by the loss of a methyl radical.

#### 2.2.3. Metabolites and Pathways of DH 

**Figure 5:**
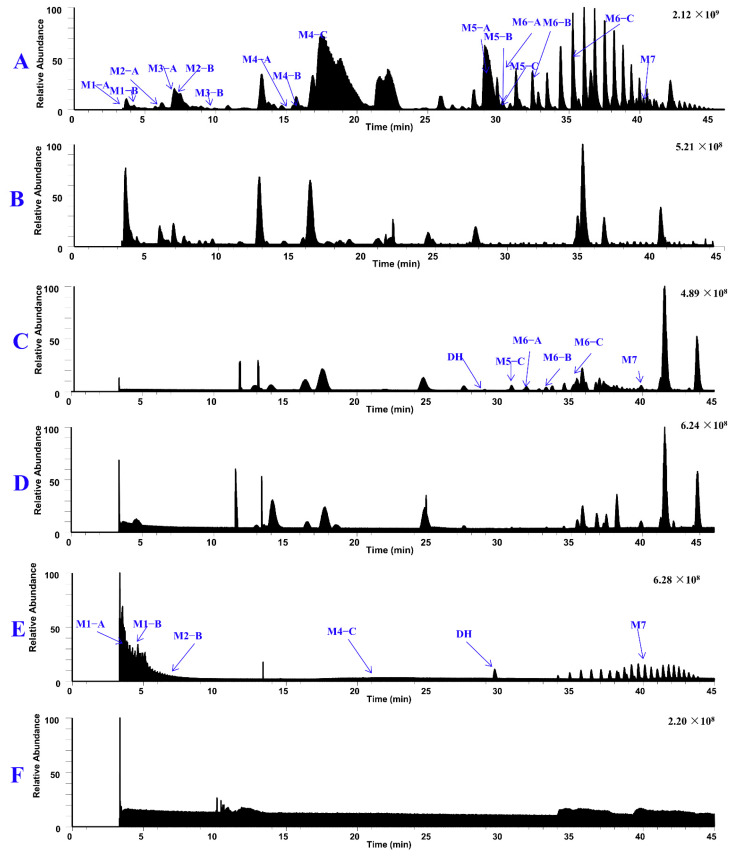
The total ion chromatograms of DH and its metabolites in pooled urine samples (**A**), blank urine samples (**B**), pooled feces samples (**C**), blank feces samples (**D**), pooled plasma samples (**E**), and blank plasma samples (**F**).

**Figure 6:**
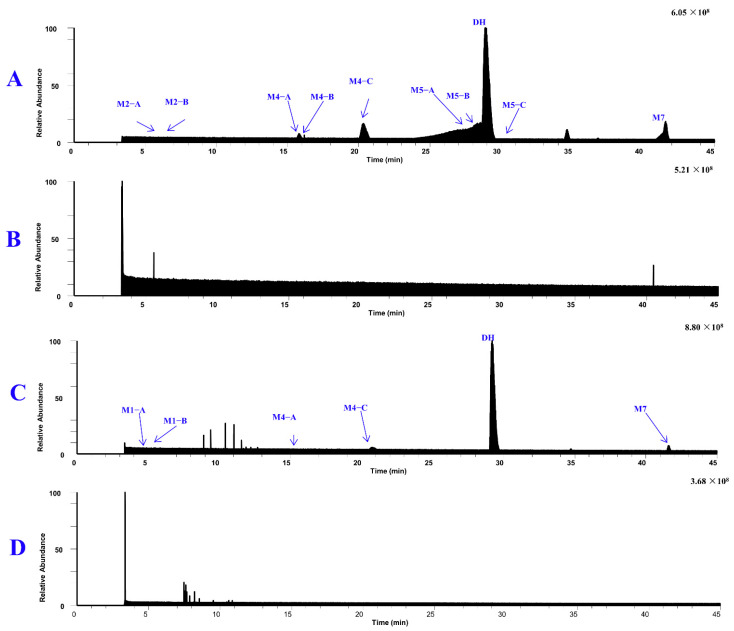
The total ion chromatograms of DH and its metabolites in pooled liver microsome phase I samples (**A**), blank liver microsome phase I samples (**B**), pooled liver microsome phase II samples (**C**), and blank liver microsome phase II samples (**D**).

**Figure 7:**
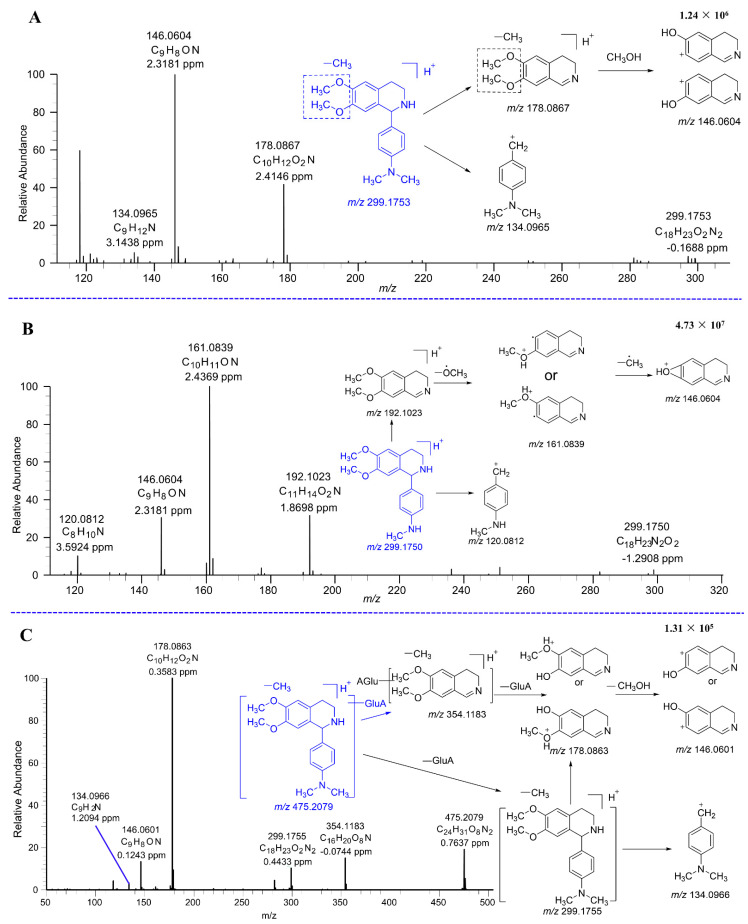
The MS/MS spectra of M4–A/M4–B (**A**), M4–C (**B**), and M1–A/M1–B (**C**).

Figure 5 displays the total ion chromatograms of DH and its metabolites in urine, plasma, and feces. Figure 6 displays the total ion chromatograms of DH and its metabolites in liver microsomes. A total of 16 metabolites were tentatively identified throughout this study, with 12 tentatively identified as phase I metabolites and 4 as phase II metabolites. Detailed information regarding accurate mass measurements and key fragmental ions of DH and its metabolites is shown in Table 1. The fragmentation processes of M1–A/M1–B, M4–A/M4–B, and M4–C can be found in Figure 7.

**Figure 8:**
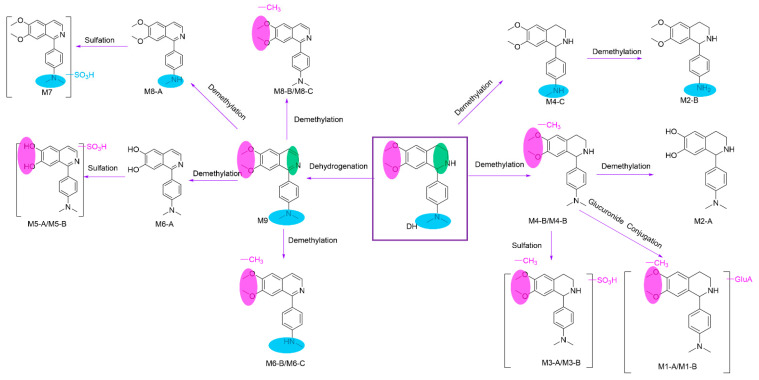
The proposed metabolic pathways of DH in rats.

M1–A and M1–B were the glucuronidated metabolites of demethylated DH. The major daughter ions were observed at *m*/*z* 354.1183, 299.1755, 178.0863, 146.0601, and 134.0966. The daughter ion 299.1755 was produced by the loss of the glucuronide group from the M1–A/M1–B. Subsequently, the daughter ions 178.0863, 146.0601, and 134.0966 were similar to M4–A/M4–B. The fragment ion *m*/*z* 354.1183 was generated by the loss of a 4′-dimethylaminophenyl from M1–A/M1–B. Figure 8 shows the potential metabolic pathways of DH. The primary metabolic reactions identified were demethylation, dehydrogenation, glucuronidation, and sulfation.

### 2.3. UHPLC-MS/MS Method Validation

#### 2.3.1. Selectivity

**Figure 9:**
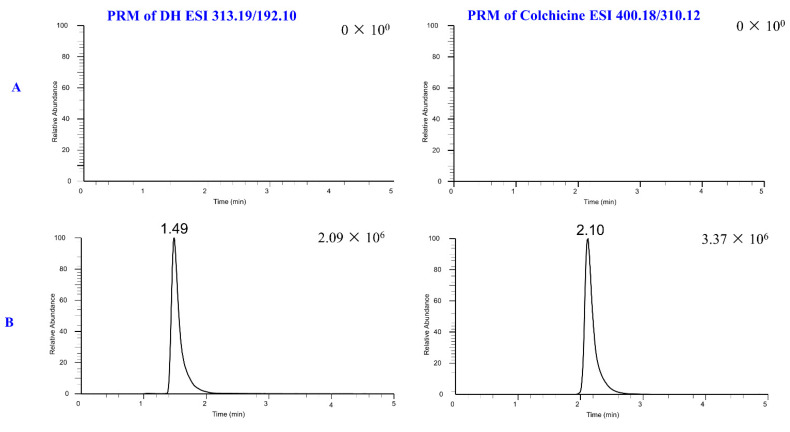

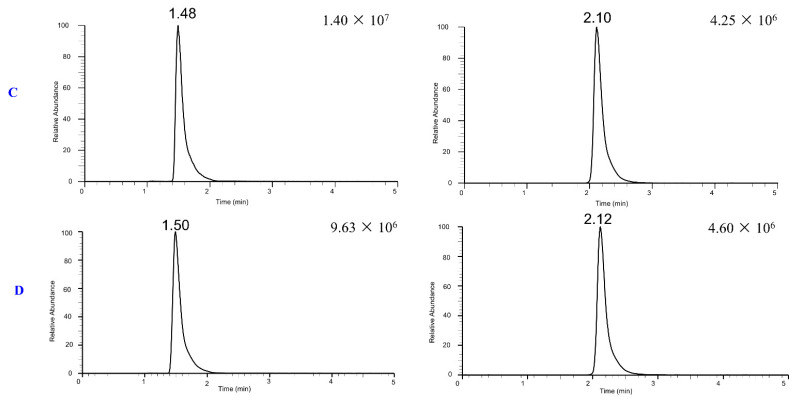
The PRM extracted ion chromatogram of DH and IS in rat plasma: blank rat plasma (**A**); blank rat plasma spiked with 100 ng/mL and IS (**B**); an incurred sample after oral administration of DH at 1.5 h (**C**); an incurred sample after intravenous administration of DH at 1.0 h (**D**).

Six rats were used to obtain blank plasma samples. Mixed plasma samples were created to prepare DH (100 ng/mL) and IS samples (300 ng/mL). During testing, DH and IS exhibited retention times of 2.10 and 1.49 min, respectively. To determine method specificity, blank plasma samples from the six rats were analyzed, and no interference peaks were detected at the retention time of DH or the IS. The results are shown in Figure 9.

#### 2.3.2. Calibration Curves

We then conducted a linear regression of the calibration curves using least squares. Over the range of 1–1000 ng/mL, good linearity (r^2^ = 0.995) was observed for DH using the regression Equation (1):

Y = 0.297674 + 0.0249991X(1)

The deviation of all back-calculated concentrations from the nominal values met the acceptance criteria for precision and accuracy (15%), so the results were deemed adequate.

#### 2.3.3. Accuracy and Precision

To assess the precision and accuracy of the method, intra-day and inter-day variability tests were conducted on plasma samples at four QC concentrations of 1, 10, 100, and 500 ng/mL. These tests aimed to determine the suitability of the technique for analyzing rat plasma samples. Precision and accuracy were defined by calculating the relative standard deviation (%RSD) and relative error (%RE), respectively. As observed in Table 2, the %RSD and %RE figures were both below 15%, indicating satisfactory levels of precision and accuracy.

#### 2.3.4. Recovery and Matrix Effect

The mean recovery for DH was calculated at 10,100, and 500 ng/mL concentrations. It ranged from 85.22–89.22%. The mean recovery for IS was 86.95%, and the results are presented in Table 3.

Some common extracts of the sample may affect the ionization efficiency of the target compound, and this effect can be observed in the instrument response, where the signal of the compound was enhanced or, more commonly, suppressed. In Table 3, the matrix effect of DH ranged from 84.42–87.55%. This range was less than 100%, indicating that these co-extracts inhibited the ionization of DH.

#### 2.3.5. Stability

Three concentrations were used to test the stability of plasma samples in a variety of circumstances. The outcomes demonstrated that when the rat plasma samples were kept at room temperature, they were stable for at least 12 h. Its accuracy range was 97.46–109.44%. When samples were stored at −80 °C, the accuracy of the samples ranged from 99.44% to 109.22% at three concentrations on days 1, 7, and 15. The prepared QC samples were placed in the autosampler and injected at 4, 8, and 12 h. The accuracy was calculated to be in the range of 103.34–110.57%. The prepared stability samples were stored at −80 °C and the accuracy range was measured from 97.37% to 104.56% after three sequential freeze–thaw cycles. All the above results are summarized in Table 4.

### 2.4. Pharmacokinetic (PK) Analysis and PK Parameter

**Figure 10:**
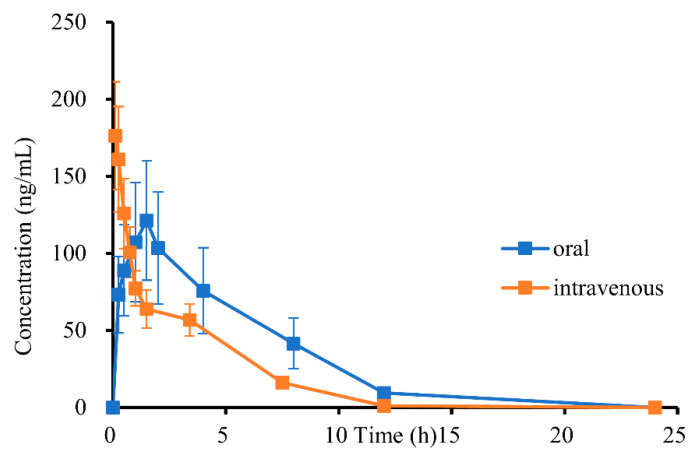
Mean plasma concentration time curves of DH in rats after oral 19.2 mg/kg (n = 6, mean ± SD) and intravenous administration 1.9 mg/kg (n = 6, mean ± SD), respectively.

The validated method was employed to simultaneously quantify DH in rat plasma and was found to be successful. Figure 10 displays the plasma concentration–time curves of DH after administration.

The non-compartmental analysis method was employed to calculate the primary pharmacokinetic parameters of DH, which are presented in Table 5. Following oral administration, the maximum concentration (C_max_) of DH was observed to be 121.35 ± 42.41 ng/mL at a Tmax of 1.50 ± 0 h. As time progressed, the concentration of DH decreased with a half-life (T_1/2_) of 2.96 ± 0.32 h. Upon intravenous administration, the clearance (CL) and apparent volume of distribution (V_d_) values were 3859.65 ± 301.55 mL/h/kg and 15,064.60 ± 2159.75 mL/kg, respectively. It appears that DH has a particular tissue distribution (15,064.60 ± 2159.75 mL/kg) in rats since the plasma distribution volume of the substance was significantly higher than the overall plasma volume of the rats [34]. The average absorption time of DH was calculated to be 1.58 h by subtracting the mean residence time of injection from oral administration. This shows that both DH absorption and elimination half-lives are reasonable, indicating DH is a good lead compound. The oral bioavailability of DH was 16.11%. This shows that DH can be made safer by improving its bioavailability and reducing the dose administered through formulation technology.

## 3. Materials and Methods

### 3.1. Reagents and Chemicals

The S. Yu. Yunusov Institute of the Chemistry of Plant Substances, Academy of Sciences of the Republic of Uzbekistan (Tashkent, Uzbekistan), provided the DH (98.5%). Colchicine (98.0%, internal standard (IS)) was purchased from the Shanghai Hong Yong Biotechnology firm (Shanghai, China). Wuhan purity biotechnology provided the blank rat plasma (Wuhan, China). Methanol, acetonitrile, and formic acid of UHPLC-MS quality were bought from Thermo Fisher Scientific (Bremen, Germany). Tianjin Xin Platinum Chemical Company (Tianjin, China) supplied ethanol, while Watsons (Guangzhou, China) provided distilled water. The Kolliphor (PEG–35, CAS, 61791–12–6) originated from Sigma-Aldrich (St. Louis, MO, USA).

### 3.2. Animals, Dosing, and Sample Collection

The Xinjiang Medical University (Xinjiang, China) provided twelve male Sprague–Dawley (SD) rats (weight 200 ± 20 g). The Animal Experimentation Ethics Committee of Xinjiang Medical University gave its approval to all animal experiments. DH was created using a mixture consisting of 2% ethanol, 8% Kolliphor, and 90% distilled water. Twelve rats were selected randomly and divided into two groups. Before administering the oral dose, the rats were fasted for 12 h but were allowed access to water. Each rat in the oral group was orally given 19.2 mg/kg of DH. Blood samples were collected from their ophthalmic veins at 0, 0.25, 0.5, 1, 1.5, 2, 4, 8, 12, and 24 h for the oral study. In the intravenous group, each rat was given an intravenous tail injection of 1.9 mg/kg of DH. Blood was collected from their ocular veins at 0, 0.1, 0.25, 0.5, 0.75, 1, 1.5, 3.4, 7.5, 12, and 24 h for further analysis. The collected plasma was thereafter subjected to centrifugation (14,000 rpm) for 10 min in a 1.5 mL polyethylene collection tube with EDTA. Following centrifugation, the supernatant was stored in a refrigerator at –80 °C.

### 3.3. UHPLC-MS/MS Conditions

Many different mass analyzers have been used for the identification of metabolites of medicines [35]. In our study, the UHPLC-Q-Orbitrap HRMS technique was used for the characterization of components in metabolites of DH. With a resolution as high as 140,000, Q-Orbitrap demonstrates a substantially greater mass-resolving power when compared to regular Q-TOF. The Orbitrap’s strong, straightforward mass-axis calibration is one of its strengths. Furthermore, compared to TOF baselines, Orbitrap baselines seem to be somewhat smoother [36].

Rat plasma samples were assessed for DH using a Thermo Fisher Scientific Q Exactive Plus Orbitrap MS and a Dionnex Ultimate 3000 RSL nano System (Thermo Fisher Scientific, Bremen, Germany). Chromatographic separation was performed with a pre-column (1.7 µm) and an ACQUITY UHPLC BEH C_18_ column (2.1 × 75 mm, 1.7 µm). The procedure utilized a 10 µL injection volume and was carried out for 5 min at 0.2 mL/min isocratic (70%A (0.1% formic acid in water): 30%B (acetonitrile)). The temperature of the RS autosampler was maintained at 10 °C, and the column was set at 35 °C. Positive electrospray ionization (ESI) and the Parallel Reaction Monitor (PRM) mode were employed to identify DH and the IS. AGC targets were set at 1 × 10^6^, the maximum iteration time at 100 ms, the spray voltage at 3.2 kV, the capillary temperature at 320 °C, the S-lens RF level at 55, and 10 and 38 for the IS of the high collision dissociation cell of DH were the MS parameters used. The PRM transitions for DH and IS were 313.19/192.10 (*m*/*z*) and 400.18/310.12 (*m*/*z*), respectively.

For the analysis of DH metabolites, the same UHPLC-MS/MS system was used for the plasma analysis, and the ACQUIT UHPLC BEH C_18_ column was also utilized. Mobile phase A consisted of 0.1% formic acid in water (*v*/*v*), while mobile phase B comprised acetonitrile. The flow rate was maintained at 0.2 mL/min, with a 10 µL injection volume. The column temperature was kept constant at 35 °C, and the RS autosampler temperature was maintained at 10 °C. The gradient elution was as follows: 0–3 min(5%B), 3–11 min (7%B), 11–18 min (7%B), and 18–45 min (30%B). A full MS/dd MS^2^ mode in positive heated electrospray ionization (HESI) with 140,000 (for MS) and 17,500 (for MS/MS) resolutions was used for the Q–Exactive Plus Orbitrap MS. The compounds identified in full MS/dd MS^2^ mode will be scanned using PRM scanning. The following MS parameters were employed: AGC target, 1 × 10^6^; Maximum IT, 100 ms; Spray voltage, 3.2 kV; Capillary temp, 320 °C; S-lens RF level, 55; HCD Energy 20, 40, and 60.

### 3.4. Preparation of Rat Plasma Solutions

#### 3.4.1. Preparation of Calibration Standards and Internal Standard Solutions

To create calibration standard and IS primary stock solutions of DH, 0.1 mL of dimethyl sulfoxide was used to dissolve DH, and this solution was then prepared using 30% acetonitrile water to achieve a concentration of 1.0 mg/mL. IS was dissolved in water to give a final standard solution of 1.0 mg/mL. To develop the calibration curve, 10 µL of the appropriate dilution standard solution (ranging from 10 to 10,000 ng/mL) and 3000 ng/mL of colchicine (the IS) were added to an aliquot of rat plasma to obtain samples containing 1–1000 ng/mL of the standard and 300 ng/mL of the IS. The samples were vortexed for 3 s, and then 300 μL of acetonitrile was added to eliminate the protein and vortexed for an additional 1 min. The mixture was centrifuged for 7 min at 14,000 rpm. The mixture supernatant was collected and blown dry at 40 °C under nitrogen. Blow-dried samples were re-dissolved in 100 μL of 30% acetonitrile in water and centrifuged for 7 min.

#### 3.4.2. Preparation of Quality Control (QC) Solutions

DH was added to the blank plasma to produce concentrations of 1, 10, 100, and 500 ng/mL QC samples. Then, 10 µL DH was added to blank plasma and vortexed for 3 s. Next, 10 µL IS was added to the plasma and vortexed for 3 s, while 300 µL of acetonitrile was added to the above plasma, vortexed for 1 min, and centrifuged for 7 min. The mixture supernatant was collected and blown dry at 40 °C under nitrogen. Blow-dried samples were re-dissolved in 100 μL of 30% acetonitrile in water and centrifuged for 7 min.

#### 3.4.3. Pharmacokinetic (PK) Sample Preparation

Dissolve PK samples were stored at −80 °C at room temperature. We added 10 µL of 30% acetonitrile water and vortexed it for 3 s. We then added 10 µL of IS and vortexed it for 3 s. Then, 300 µL of acetonitrile was added to the above plasma, vortexed for 1 min, and centrifuged for 7 min. The mixture supernatant was collected and blown dry at 40 °C under nitrogen. Blow-dried samples were re-dissolved in 100 μL of 30% acetonitrile in water and centrifuged for 7 min.

### 3.5. Metabolic Study

#### 3.5.1. In Silico Metabolism Prediction

XenoSite [30] performs deep learning of neural networks to predict the possible metabolic sites of DH. The physical and chemical properties of each molecule are digitally encoded, and it is able to calculate metabolic bonds and lone pairs. Each bond is described by 404 vectors, which indicate the atom (e.g., atom identity, charge, and hybridization), bond (e.g., bond type, bond length), and molecule (e.g., molecular weight, topological polar surface area, and molar refractivity) properties [29]. XenoSite web page prediction models include phase I [29], epoxidation [31], quinonation [32], N-dealkylation [33], reactivity [37,38], and uridine diphosphate glucuronosyltransferases [39].

The XenoSite result is represented as a color scale with a scale from 0 to 1 (the value 1 denotes the highest level of confidence in the model’s ability to forecast the future). One can designate the outcome of XenoSite as a possibility. Additionally, it shows how confident the model is in the metabolism of an explicit section. In other words, an atom has a 90% chance of being an empirically proven site of metabolic activity if its prediction score is 0.9.

#### 3.5.2. In Vitro and In Vivo Experiments

Rat liver microsomes were used for in vitro experiments to study DH metabolism in rats more quickly and easily. After that, the cofactors of phases I and II were added to create the phase I and phase II metabolic systems. The composition of the reagent kit and the procedure can be found in our previous work [40].

For the plasma study, 100 µL of plasma was obtained from rats at 0.5, 1, 2, and 4 h after medicine administration. Then, 300 µL of acetonitrile was added to the above plasma, vortexed for 1 min, and centrifuged for 7 min. The supernatant was collected, and the remaining liquid was dried using nitrogen gas at 40 °C. The blow-dried samples were re-dissolved in 100 µL methanol, sonicated for 1 min, and centrifuged for 7 min.

Urine was collected during the period spanning from 0 to 48 h following administration, with each period’s combined urine comprising 10 mL and 30 mL of acetonitrile added thereto. After a 10 min interval of sonication, the urine samples were transferred into 1.5 mL centrifuge tubes, vortexed for 1 min, and then centrifuged at a speed of 14,000 rpm for 10 min. Successively, the supernatant was merged and subjected to drying by nitrogen gas at 40 °C. The blow-dried samples were re-dissolved in 100 µL of methanol, sonicated for 1 min, and centrifuged for 7 min.

For the fecal study, feces were collected within 0–48 h following administration, dried to powder at room temperature in a shaded area, and combined to yield 8 mL per period, to which 40 mL of acetonitrile was added. Following 30 min of sonication, the samples were transferred to 1.5 mL centrifuge tubes and centrifuged at 14,000 rpm for 10 min. The supernatant was collected and dried with nitrogen at 40 °C before analysis via UHPLC-MS/MS. Before analysis, the blow-dried samples were re-dissolved in 100 µL of methanol, sonicated for 1 min, and centrifuged for 7 min.

Compound Discovery 3.3 was used for data processing. The MS spectrometric features mainly include retention time, exact mass, peak intensity, and fragment ions. The data processing mainly included selecting spectra, aligning retention times, creating pattern traces, creating fish traces, generating expected compounds, finding expected compounds, grouping expected compounds, fish scoring, and marking background compounds. The mass tolerance is less than 5 ppm.

### 3.6. Method Validation

The proposed UHPLC-MS/MS method was verified for specificity, accuracy, precision, recovery, matrix effects, and stability according to US FDA guidelines as described below [41].

Although acetonitrile precipitates most of the protein, plasma samples contain phospholipids, metabolites, etc., that interfere with the detection of DH and IS. Specificity was mainly examined at DH and IS retention times without interference peaks in blank plasma. In addition, samples tested after administration should not show interfering peaks at the DH and IS peak times. The standard curves were made up of 8 nonzero analyte points, and the linearity was tested. The correlation of peak area (analyte/IS) with the labeled plasma concentration was plotted for regression analysis. This analyte was predicted to have a correlation coefficient (r^2^) of more than 0.99 with the corresponding standard curve. The relative standard deviation (RSD) and relative error (RE) values were calculated by measuring six repeated QC samples on the same day to evaluate the intra-day precision and accuracy. Inter-day precision and accuracy were characterized by RSD and RE, respectively. The recoveries were determined by examining the ratio of the peak areas of DH before and after extraction. The matrix effect is the ratio of the peak area of DH added to plasma and DH dissolved in the solvent. The stability evaluation should guarantee that the concentration of the analyte remains unchanged throughout the sample preparation and analysis process, as well as during storage. Stability studies include long-term storage stability at −80 °C (1, 7 and 15 days); repeated freeze–thaw stability; autosampler stability; room temperature stability; and long-term storage stability of stock solutions.

### 3.7. Pharmacokinetic (PK) Study

Twelve male SD rats, weighing 200 ± 20 mg, were used to perform PK studies on DH. SD rats fasted for 12 h before administration, but the water was freely available. Normal drinking and eating resumed four hours after administration. To study the bioavailability and PK parameters of DH, rats were randomly divided into two groups. The dose administered was 1.9 mg/kg for the intravenous group and 19.2 mg/kg for the oral administration group. Oral bioavailability was calculated using Equation (2) as follows:

Oral bioavailability (%) = (AUCp.o. × Dosei.v./AUCi.v. × Dosep. o.) × 100%(2)

### 3.8. Data Analysis

The Q Exactive Plus Orbitrap MS data were acquired using the Thermo Xcalibur 4.2. software (Thermo Fisher Scientific, Waltham, MA, USA). The Qual Browser was used to extract ion chromatograms for screening candidate metabolites with a mass tolerance of 5 ppm and a mass precision of 4 decimals. In addition, MS/MS fragment ions were extracted for structural confirmation of candidate metabolites. The Qual Browser was used for the quantification of DH in rat plasma. Pharmacokinetic parameters were processed using the non-compartmental pharmacokinetic data analysis software program of MaS Studio 1.5.2.14 stable (Shanghai Boca Pharmaceutical Technology Co., Shanghai, China). Finally, the results were described as arithmetic mean ± SD.

## 4. Conclusions

This study will help us better understand the metabolic process of DH and its pharmacokinetic characteristics in rats. Regarding in silico prediction, DH was less reactive with glutathione, proteins, and DNA. A total of 16 metabolites were discovered and tentatively identified from the plasma, urine, feces, and liver microsomes of rats, including 12 phase I and 6 phase II metabolites. The proposed metabolic pathways for DH were mainly demethylation, dehydrogenation, glucuronidation, and sulfation. The glucuronide metabolites of DH were not found either in vitro or in vivo, even though there was a high probability of glucuronide in silico. This may be attributed to the species differences between humans and rats. The method has been effectively applied to a pharmacokinetic study for the quantification of DH in plasma. As a consequence, the absolute bioavailability of DH was determined to be 16.11% when non-compartmental analysis was used to determine the pharmacokinetic characteristics of intravenous and oral administrations of DH. The calculation of the average DH absorption time, which was determined to be roughly 1.58 h, indicates that DH has a decent absorption rate. Although DH bioavailability is not ideal, it can be improved by nanoformulations or liposomes. The reasonable absorption time and elimination half-life of DH provide a positive sign that it could be a better medicine candidate.

## Figures and Tables

**Table 1:** Metabolites characterization of DH in rats.

Metabolites	*t*_R_ (min)	Formula (M+H^+^)	Observed Mass (*m*/*z*)	Calculated Mass	Error (ppm)	Fragmention	Transformations	Rat and LM*
DH	28.88	C_19_H_25_N_2_O_2_	313.1911	313.1911	0.2	192.1023 (71), 161.0840 (100), 146.0604 (30), 134.0967 (8)	Parent	P*, U*, F*, LM*
M1–A	3.65	C_24_H_31_N_2_O_8_	475.2081	475.2075	1.2	354.1183 (44), 178.0864 (100), 146.0600 (48), 134.0966 (5)	Demethylation, Glucuronide Conjugation	P*, U*, LM*
M1–B	4.83	C_24_H_31_N_2_O_8_	475.2081	475.2075	1.2	354.1182 (36), 178.0865 (100), 146.0603 (55), 134.0960 (5)	Demethylation, Glucuronic Conjugation	P*, U*, LM*
M2–A	5.61	C_17_H_21_N_2_O_2_	285.1598	285.1598	0.2	283.1449 (5), 164.0710 (97), 146.0604 (100), 134.0964 (6), 122.0969 (54)	Demethylation	U*, LM*
M2–B	6.93	C_17_H_21_N_2_O_2_	285.1598	285.1598	0.3	192.1026 (67), 178.0866 (28), 161.0836 (100), 146.0635 (62),	Demethylation,	P*, U*, LM*
M3–A	6.51	C_18_H_23_N_2_O_5_S	379.1316	379.1322	−1.6	258.0431 (32), 178.0863 (96), 146.0601 (100), 122.0967 (15)	Demethylation, Sulfation	U*
M3–B	9.84	C_18_H_23_N_2_O_5_S	379.1323	379.1323	0.2	258.0431 (24), 178.0864 (100), 146.0602 (98), 122.0967 (14)	Demethylation, Sulfation	U*
M4–A	15.05	C_18_H_23_N_2_O_2_	299.1753	299.1754	−0.3	178.0867 (100), 146.0604 (98), 134.0965 (5)	Demethylation	U*, LM*
M4–B	15.71	C_18_H_23_N_2_O_2_	299.1754	299.1754	−0.2	178.0866 (100), 146.0604 (90), 134.0969 (7)	Demethylation	U*, LM*
M4–C	19.47	C_18_H_23_N_2_O_2_	299.1750	299.1754	−1.3	192.1023 (72), 161.0839 (100), 146.0604 (28), 120.0812 (13)	Demethylation	P*, U*, LM*
M5–A	26.68	C_17_H_17_N_2_O_2_	281.1283	281.1285	−0.45	266.1046 (22), 238.1101 (24)	Dehydrogenation, Demethylation	U*, LM*
M5–B	29.29	C_17_H_17_N_2_O_2_	281.1288	281.1285	1.0	266.1054 (27), 238.1105 (23)	Dehydrogenation, Demethylation	U*, LM*
M5–C	30.44	C_17_H_17_N_2_O_2_	281.1289	281.1285	1.3	266.1054 (40), 238.1105 (39)	Dehydrogenation, Demethylation	U*, F*, LM*
M6–A	30.99	C_18_H_19_N_2_O_2_	295.1446	295.1441	1.5	280.1209 (22), 252.1283 (20)	Dehydrogenation, Demethylation	F*
M6–B	34.13	C_18_H_19_N_2_O_2_	295.1447	295.1441	1.2	280.1211 (20), 252.1263 (17)	Dehydrogenation, Demethylation	U*, F*
M6–C	35.58	C_18_H_19_N_2_O_2_	295.1440	295.1441	−0.31	280.1202 (30), 252.1256 (25)	Dehydrogenation, Demethylation	U*, F*
M7	40.10	C_19_H_21_N_2_O_2_	309.1602	309.1598	1.4	293.1288 (9), 265.1340 (16)	Dehydrogenation	P*, U*, F*, LM*

F*, feces; U*, urine; P*, plasma; LM*, Liver microsome.

**Table 2:** The validation parameters: intra-day and inter-day accuracies and precisions of DH in rat plasma (n = 6).

Analytes	Nominal Concentration (ng/mL)	Intra-Day Mean	RSD (%)	RE (%)	Inter-Day Mean	RSD (%)	RE (%)
DH	1	1.08	7.41	8.00	1.02	8.16	2.00
	10	9.51	6.03	−4.90	9.93	7.84	−0.70
	100	98.38	5.64	−1.62	104.5	5.44	4.50
	500	497.00	3.91	−0.60	540.82	2.49	8.00

**Table 3:** The validation parameters: recovery and matrix effect of DH and the IS in rat plasma.

Analytes	Nominal Concentration (ng/mL)	Recovery		Matrix Effect	
		Mean (%)	RSD (%)	Mean (%)	RSD (%)
DH	10	89.22	12.67	87.55	6.30
	100	87.26	4.51	88.16	2.97
	500	85.43	0.97	84.42	1.85
IS	300	86.95	1.32	85.47	4.75

**Table 4:** The validation parameters: stability of DH under different conditions.

Analytes	Nominal Concentration (ng/mL)	Accuracy								
DH		4 h	8 h	12 h	1 day	7 days	15 days	Stock solution	Room temper	Thaw
	10	110.30	108.80	110.57	109.12	108.43	106.68	104.96	109.44	100.00
	100	106.65	106.69	103.81	103.58	106.23	101.13	99.49	101.36	104.56
	500	105.03	103.54	103.34	99.17	105.59	94.99	96.24	97.46	97.37

**Table 5:** The main pharmacokinetic parameters of DH in rats after intravenous and oral administration. (n = 6, mean ± standard deviation).

Parameters	Unit	P.O. (19.2 mg/kg)	I.V. (1.9 mg/kg)
AUC_0–t_ *	h* (ng/mL)	707.28 ± 264.72	439.08 ± 30.11
AUC_0–∞_ *	h* (ng/mL)	748.44 ± 278.98	494.64 ± 36.40
MRT_0–t_ *	h	4.07 ± 0.34	2.49 ± 0.33
MRT_0–∞_ *	h	4.76 ± 0.40	3.56 ± 0.26
T_max_ *	h	1.50 ± 0.00	—
T_1/2_ *	h	2.96 ± 0.32	2.79 ± 0.34
C_max_ *	ng/mL	121.35 ± 42.41	183.37 ± 33.56
CL *	mL/h/kg	30,148.65 ± 15,354.27	3859.65 ± 301.53
V_d_ *	mL/kg	126,728.09 ± 56,867.09	15,514.58 ± 2159.74
F_abs_ (%) *	16.11		

AUC_0–t_ * (AUC_0–∞_), area under the analyte concentrations versus time curve from time 0 to t h (∞); MRT_0–t_ * (MRT_0–∞_), Mean residence time at time 0–t (∞); T_max_ *, the time of maximum concentration; C_max_ *, maximum concentration; T_1/2_ *, terminal half-life; CL *, clearance; V_d_ *, apparent volume of distribution; F_abs_ (%) *, absolute bioavailability.

## Data Availability

Data are contained within the article.
